# Pre-hospital delay in patients with myocardial infarction: an observational study in a tertiary care hospital of northern Bangladesh

**DOI:** 10.1186/s12913-020-05505-x

**Published:** 2020-07-09

**Authors:** Abdur Rafi, Zahidus Sayeed, Papia Sultana, Saw Aik, Golam Hossain

**Affiliations:** 1 Rajshahi Medical College, Rajshahi, 6100 Bangladesh; 2 Department of Statistics, University of Rajshahi, Rajshahi, Bangladesh; 3 Department of Orthopaedic Surgery, University of Malaya, National Orthopaedic Centre of Excellence for Research and Learning (NOCERAL), Kuala Lumpur, Malaysia

**Keywords:** Pre-hospital delay, Myocardial infarction, In-hospital mortality, Bangladesh

## Abstract

**Background:**

Delayed hospital presentation is a hindrance to the optimum clinical outcome of modern therapies of Myocardial infarction (MI). This study aimed to investigate the significant factors associated with prolonged pre-hospital delay and the impact of this delay on in-hospital mortality among patients with MI in Northern Bangladesh.

**Methods:**

This cross sectional study was conducted in December 2019 in cardiology ward of a 1000-bed tertiary care hospital of Bangladesh. Patients admitted in the ward with the diagnosis of myocardial infarction were included in the study. Socio demographic data, clinical features and patients’ health seeking behavior was collected in a structured questionnaire from the patients. Median with interquartile range (IQR) of pre hospital delay were calculated and compared between different groups. Chi-square (χ^2^) test and binary logistic regression were used to estimate the determinants of pre-hospital delay and effect of pre-hospital delay on in-hospital mortality.

**Results:**

Three hundred thirty-seven patients was enrolled in the study and their median (IQR) pre-hospital delay was 9.0 (13.0) hours. 39.5% patients admitted in the specialized hospital within 6 h. In logistic regression, determinants of pre-hospital delay were patients age (for < 40 years aOR 2.43, 95% CI 0.73–8.12; for 40 to 60 years aOR 0.44, 95% CI 0.21–0.93), family income (for lower income aOR 5.74, 95% CI 0.89–37.06; for middle income aOR 14.22, 95% CI 2.15–94.17), distance from primary care center ≤5 km (aOR 0.42, 95% CI 0.12–0.90), predominant chest pain (aOR 0.15, 95% CI 0.05–0.48), considering symptoms as non-significant (aOR 17.81, 95% CI 5.92–53.48), referral from primary care center (for government hospital aOR 4.45, 95% CI 2.03–9.74; for private hospital OR 98.67, 95% CI 11.87–820.34); and not having family history of MI (aOR 2.65, 95% CI 1.24–5.71) (R2 = 0.528). Risk of in-hospital mortality was almost four times higher who admitted after 6 h compared to their counterpart (aOR 0.28, 95% CI 0.12–0.66); (*R*^2^ = 0.303).

**Conclusion:**

Some modifiable factors contribute to higher pre-hospital delay of MI patients, resulting in increased in-hospital mortality. Patients’ awareness about cardiovascular diseases and improved referral pathway of the existing health care system may reduce this unexpected delay.

## Background

Cardiovascular disease is a major cause of death globally. More than three quarters of these deaths occurred in lower-and middle-income countries [1]. Coronary artery disease, especially myocardial infarction (MI) is one of the leading cardiovascular diseases with a high mortality rate. Treatment of acute MI with modern reperfusion therapy like percutaneous coronary intervention (PCI) and thrombolytic therapy, is exclusively time dependent [2]. Delay in treatment has been associated with higher rate of morbidity and mortality [3, 4]. Therefore, reducing the delay before initiation of reperfusion therapy is an important strategy to improve the success rate of treatment for this condition.

Over the last few decades, Bangladesh, a lower/middle-income country in South-East Asian region is at an epidemiological transition with increasing death rates from non-communicable diseases [5]. However, the existing health care system which was designed for the prevention and treatment of communicable diseases is not prepared for these emerging challenges. As a result, advanced treatment for chronic conditions is not widely available. Reperfusion therapy for MI is mainly offered by tertiary level or specialized hospitals, and thrombolytic therapy is the primary option since PCI facilities are still readily available. For this reason, pre-hospital delay (the time between the onset of MI symptoms and arrival at these hospitals) for patients with acute MI may be considerably long.

To ensure optimum benefit, some clinicians recommended that thrombolytic therapy (intra-venous Streptokinase infusion) should be administered within 1 h (the golden hour) of the onset of symptoms [6], while others felt that the duration can be extended up to 6 h [7]. In other words, reducing pre-hospital delay and prompt treatment after arrival at the hospital are prerequisites for effective reperfusion therapy. In developed countries, pre-hospital delay is generally lower than those of developing countries [8, 9].

Most of the studies that were designed to identify the determinants of pre-hospital delay were conducted in developed countries. A few socio-demographic and clinical factors had been reported to be associated with longer pre-hospital delay. They included older age, female sex, low socioeconomic status, history of cardiac illness, diabetes mellitus, and hypertension. The delay was also more common in those who consulted non-medical-trained person, practiced self-treatment, downplayed the seriousness of MI, and those who have insufficient knowledge about its symptoms [10–14]. However, the socio-demographics characteristics and health care delivery system in Bangladesh is quite different, and the standard of living and awareness of health-related issues are generally low. There are no uniform primary care providers for the general public; while some patients will seek treatment from either public or private doctors for their illness, others would confide in non-qualified practitioners or drug sellers. There is no national health insurance system in Bangladesh, and most of the populations are not covered by any form of private health insurance. Although most of the patients would initially seek treatment in the government hospitals, many will eventually need to pay out-of-pocket for more sophisticated investigations, special medicine or to receive early treatment. Moreover, there is no specific referral system in Bangladesh. A patient may decide to visit the clinic of a general practitioner, outpatient or emergency department of the government or private hospital, or directly self-referred to a tertiary care or specialized hospital. For these reasons, the pre-hospital delay in patients diagnosed with acute MI would be influenced by many factors, and health-care seeking behavior would be one of the important determinants of this delay.

A recent study from Dhaka reported that rural residence, longer distance from hospital, problem with transportation, self-medication, and misinterpretation of symptoms were the predictors of late presentation to hospital their MI patients [15]. Another study from the Southern region of Bangladesh reported similar findings [16]. These findings might not be generalized for other parts of the country since the socio-demographic characteristics and health care seeking behaviors might be different. Moreover, there was no local data to verify the association between pre-hospital delay on in-hospital outcome of MI patients. We therefore designed this study to investigate the factors contributing to pre-hospital delay of MI patients in Northern region of Bangladesh, and its association with in-hospital mortality.

## Methods

### Study design and setting

This was a cross sectional descriptive study, conducted in Rajshahi Medical College Hospital (RMCH) during the month of December, 2019. RMCH is a 1000-bed tertiary care teaching hospital situated in Rajshahi, a divisional city of Northern Bangladesh serving patients from different districts of Rajshahi, Rangpur and Khulna divisions. Patients can be admitted to this hospital directly through the emergency department or by referral from different primary care hospitals. Ethical approval was obtained from the hospital ethical board before we recruit the patients and conduct the interviews.

### Sampling method

All the patients who were admitted to the Cardiology ward of RMCH with the diagnosis of acute MI within the study period would be recruited for the study. Convenient sampling technique was used to include patients who met the inclusion and exclusion criteria. The inclusion criteria were (i) patients diagnosed as acute MI between December 1 and 31, 2019, (ii) patients who were given thrombolytic therapy for the first time in RMCH and (iii) patients who gave informed written consent to participate in the study. Exclusion criteria were (i) patients who were not willing to participate in the study, (ii) patients who had other co-existing heart conditions, (iii) patients who were given thrombolytic therapy in other institution, and (iv) critically ill patients who were unable to take part in the interview or died within a short period of admission before we managed to conduct the interview.

### Sample size determination

The sample for this study was calculated using the formula: $n=\frac{z^2 pq}{d^2}$, Where z = z value for 95% conference interval, *p* = assumed prevalence of pre-hospital delay (more than 6 h), q = (1 - p) and d = precision of error for the assumed prevalence. From the previous study conducted in Southern Bangladesh [16], z = 1.96, *p* = 0.828, q = (1 - p) and d = 0.0828 (10% of p), calculated sample size was 80. However, we recruited 337 MI patients to ensure adequate power for the study.

### Data collection

A pretested structured questionnaire (additional file; questionnaire) was used for collecting data from our patients. The questionnaire was initially drafted in English based on the materials from few previous studies [9, 16, 17]. It was subsequently translated into Bangla by two independent translators using back-translation method for the convenience of the interviewers as well as the interviewees. The Bangla questionnaire was pretested on 30 MI patients who did not participated in the study for linguistic adaptation and further clarification. Three fifth-year medical students who were doing their clinical postings in cardiology department were trained to conduct the interviews. The interviews were conducted after the morning ward round, between 10 am and 12 pm, by the primary investigator and the three medical students. The questionnaire had three parts: (i) socio demographic information, (ii) clinical features (symptoms, time of onset of symptoms and diagnosis), previous medical history and cardiovascular risk factors, and (iii) health seeking behavior (primary action after onset of symptoms, time of decision to seek medical care, time and mode of admission to the hospital) and in-hospital treatment outcome (survival or death).

Information on social demographic features and presenting symptoms were obtained during the interviews in the ward, while information on clinical features, hospital admission, and treatment outcome were obtained from the medical folders of the patient.

### Outcome variable

**Fig. 1:**
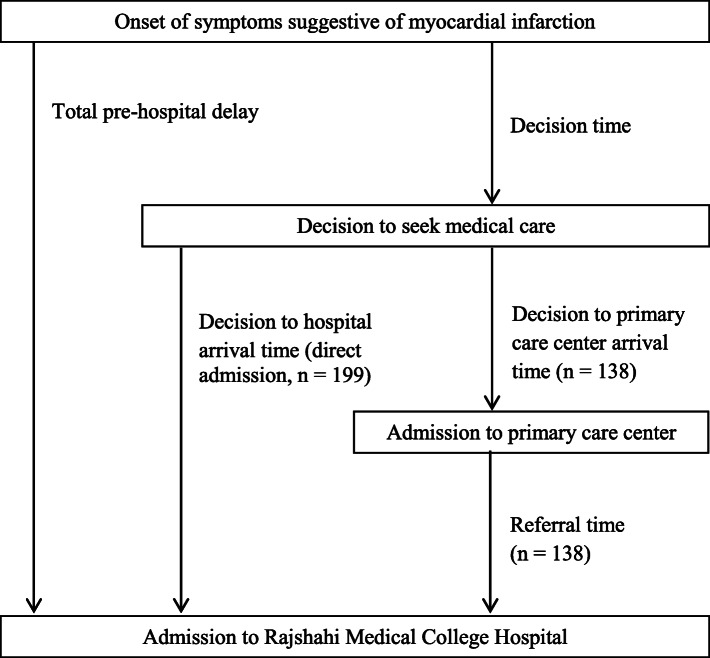
Preospital delay and definition of time intervals

Pre-hospital delay and in-hospital mortality were the two main outcome variables for the study. Pre-hospital delay was defined as the time between the onset of symptoms of MI and time of admission to RMCH. It was divided into decision time (defined as the time between onset of symptoms and decision to seek medical care) and decision to hospital arrival time. Referral time was defined as the time between admission to the first hospital and admission to RMCH. Pre-hospital delay and definition of the time intervals are shown in Fig. 1. Pre-hospital delay was classified into two classes as (i) less than 6 h, (ii) more than 6 h. We selected 6 h as the cut-off time since previous study has shown that longer pre-hospital delay was associated with increased risk of mortality [6].

### Independent variables

Independent variables were socio-demographic factors (age, sex, marital status, family income, residence) and clinical factors (distance of primary care center, mode of transport, type of diagnosed MI, clinical symptoms, behavior and actions after onset of symptoms, mode of hospital admission, previous medical history and cardiovascular risk factors). Presenting clinical symptoms were classified as predominantly chest pain symptoms (pain, ache, burn, or pressure in the chest) and predominantly other symptoms (pain in the abdomen, arm, shoulder, or neck, other than chest; and other symptoms like severe fatigue, syncope, or circulatory shock).

### Statistical analysis

Patient characteristics were presented as proportions, means, or medians. Median with interquartile range (IQR) was used for highly skewed distributions of pre-hospital delay and other delays as previously defined. Non-parametric test (Mann-Whitney U Test) was used to compare medians among different groups. Chi-square (χ^2^) test was used to find the association between outcome variables and independent variables. Multivariate logistic regression including the significant variables (*p*-value < 0.05) in univariate logistic regression was carried out to estimate the determinants associated with delayed hospital admission, this model also used to find out the association between pre-hospital delay and in-hospital treatment outcome of the patients. Statistical analysis was performed using SPSS version (IBM) 20.0.

## Results

### Patient characteristics

A total of 337 patients were recruited into the study. Their mean age (SD) was 54.37 (12.58) years. Around 75% of the patients were male and most of them were from lower- or middle-income families staying in the rural areas. Only one-third of the patients had primary care facilities within 5 km of their residence. Around 78% of them identified chest pain as the predominant clinical symptoms, and 74.5% were diagnosed as STEMI type of MI based on their ECG findings. Although almost half of the patients did suspect they had myocardial infarction or heart-related problem from the beginning, but others either misinterpreted the symptoms (20%), considered it as nothing serious (20%) or decided to wait with the hope that these symptoms would spontaneously resolve (11.6%). Only half of the patients visited qualified doctors after onset of symptoms, while others decided to consult non-qualified practitioners or self-medicate. Fifty-nine percent (59%) of these patients were admitted to RMCH directly, while others were referred from other government or private hospitals. One-third of the patients arrived in an ambulance. Many patients had history of smoking (61%), hypertension (67%), diabetes mellitus (29.4%), previous history (24.6%) or family history (34.1%) of cardiac diseases (Table 2).

### Pre-hospital delay

We noted a highly skewed distribution of pre-hospital delay with wide IQR. The median of all the delays was 9 (IQR 13) hours, where median of decision time was 2 (IQR 2.8) hours and median of decision to first admission time was 1 (1.0) hour. Median of decision to first admission time for those who directly admitted to RMCH was higher (1.5 h) than those who admitted to primary care hospital. Referral time from primary care facilities was 4 (IQR 7.5) hours, and this was longer for private hospitals compared to government hospitals (7.5 h vs 4 h) (Table 1).

**Table 1:** Total pre-hospital delay, decision time, decision to first medical admission time and referral time for patients with myocardial infarction

Characteristics	N	Time in hours, median (IQR)
Total pre-hospital delay	337	9.0 (13.0)
Decision time	337	2.0 (2.8)
Decision to first medical admission time*		
Overall	337	1.0 (1.0)
Admission to RMCH	199	1.5 (1.2)
Admission to Primary care center	138	1.0 (1.0)
Referral time*		
Overall	138	4.0 (7.5)
Private hospital	26	7.5 (17.0)
Government hospital	112	4.0 (5.75)

*Difference between different groups: Mann-Whitney U Test, *P*-value < 0.01

Only 39.5% patients were admitted to RMCH within 6 h. These patients were predominantly middle aged, from urban area, belonged to higher income families and had primary care facilities within 5 km from their residence. Gender, educational status, types of transportation and ECG characteristic (STEMI/non-STEMI) did not show any association with pre-hospital delay. We noted that type of clinical presentation, patients’ behavior, and their action after onset of symptoms significantly influenced the pre-hospital delay. Those who did not have chest pain as the predominant symptom and those who did not visit qualified practitioners had longer pre-hospital delay. Presence of cardiovascular risk factors like diabetes mellitus, history of previous chest pain, stroke and family history of coronary artery disease were associated with early hospital admission (Table 2).

**Table 2:** Characteristics of first time myocardial infarction patients according to total pre-hospital delay

Characteristics	Median Pre-hospital delay (IQR) (hours)	Total (*n* = 337)	≤6 h (*n* = 133)	> 6 h (*n* = 204)	*P*-value
Age, mean (SD)		54.37 (12.58)	53.40 (10.75)	55.01 (13.63)	0.228
Age category					
≤ 40 years	10 (12)	47 (13.9)	9 (6.8)	38 (18.6)	< 0.001
41–60 years	6 (10)	180 (53.4)	91 (68.4)	89 (43.6)	
> 60 years	11 (21)	110 (32.6)	33 (24.8)	77 (37.7)	
Sex					
Male	8 (13)	254 (75.4)	103 (77.4)	151 (74.0)	0.476
Female	12 (18)	83 (24.6)	30 (22.6)	53 (26.0)	
Marital status					
Married	8 (13)	294 (87.2)	112 (84.2)	182 (89.2)	0.178
Single/widowed	9 (13)	43 (12.8)	21 (15.8)	22 (10.8)	
Educational status					
None/primary	10.5 (23)	224 (66.5)	82 (36.6)	142 (63.4)	0.111
Secondary/Higher secondary	9 (15)	68 (20.2)	27 (39.7)	41 (60.3)	
University graduate	6 (7)	45 (13.4)	24 (53.3)	21 (46.7)	
Family income					
Lower (< BDT 15000)	8 (15)	210 (62.3)	85 (63.9)	125 (61.3)	< 0.001
Middle (BDT 15000 – BDT 30000)	10 (12)	102 (30.3)	30 (22.6)	72 (35.3)	
Higher (> BDT 30000)	4 (8)	25 (7.4)	18 (13.5)	7 (3.4)	
Residence					
Rural	9 (13)	233 (69.1)	82 (61.7)	151 (74.0)	0.016
Urban	6.5 (12)	104 (30.9)	51 (38.3)	53 (26.0)	
Distance of primary care center					
≤ 5 km	7.5 (12)	238 (70.6)	109 (82.0)	129 (63.2)	< 0.001
> 5 km	11 (19)	99 (29.4)	24 (18.0)	75 (36.8)	
Mode of transport					
Ambulance	8 (13)	96 (28.5)	40 (30.1)	56 (27.5)	0.602
General transport	8 (14)	241 (71.5)	93 (69.9)	148 (72.5)	
Diagnosis					
STEMI	8 (14)	251 (74.5)	100 (75.2)	151 (74.0)	0.810
Non-STEMI	8 (18)	86 (25.5)	33 (24.8)	53 (26.0)	
Predominant clinical symptom					
Chest pain	6.5 (8)	262 (77.7)	125 (94.0)	137 (67.2)	< 0.001
Symptoms other than chest pain	28 (38)	75 (22.3)	8 (6.0)	67 (32.8)	
Behavior after onset of symptoms					
Misinterpreting the nature of pain	6 (8)	67 (19.9)	30 (22.6)	37 (18.1)	< 0.001
Did not consider the symptoms to be serious	16 (20)	67 (19.9)	6 (4.5)	61 (29.9)	
Waited to see symptoms would going	45 (76)	39 (11.6)	0 (0.0)	39 (19.1)	
Suspected as MI	5 (5)	164 (48.7)	97 (72.9)	67 (32.8)	
First medical action after onset of symptoms					
Visiting qualified doctor	8 (9)	188 (55.8)	89 (66.9)	99 (48.5)	0.003
Visiting non-qualified practitioner	12 (26)	85 (25.2)	23 (17.3)	62 (30.4)	
Self-medication	8 (19)	64 (19.0)	21 (15.8)	43 (21.1)	
Mode of admission					
Direct admission	8 (13)	199 (59.1)	90 (67.7)	109 (53.4)	0.003
Referred from government hospital	8 (12)	112 (33.2)	40 (30.1)	72 (35.3)	
Referred from private hospital	15 (24)	26 (7.7)	3 (2.3)	23 (11.3)	
Medical history/ risk factors					
Smoking	8 (15)	206 (61.1)	82 (61.7)	124 (60.8)	0.873
Sedentary lifestyle	8 (12)	111 (32.9)	44 (33.1)	67 (32.8)	0.964
Diabetes mellitus	6 (19)	99 (29.4)	49 (36.8)	50 (24.5)	0.015
Hypertension	8 (15)	225 (66.8)	92 (69.2)	133 (65.2)	0.449
Previous history of chest pain	6 (9)	83 (24.6)	43 (32.3)	40 (19.6)	0.008
Previous history of stroke	31 (19)	9 (2.7)	0 (0.0)	9 (4.4)	0.013
Family history of myocardial infarction	6 (8)	115 (34.1)	58 (43.6)	57 (27.9)	0.003

Logistic regression model demonstrated that pre-hospital delay was shorter by 56% among patients aged 41–60 years (aOR = 0.44, 95% CI: 0.21–0.93; *p* < 0.05) compared to aged patients (age > 60 years). Pre-hospital delay was 5.74 and 14.22 times longer among patients living in lower income families (aOR = 5.74, 95% CI: 0.89–37.06; *p* = 0.066) and middle income families (aOR = 14.22, 95% CI 2.15–94.17; *p* < 0.01) when compared those from higher income families. Pre-hospital delay was shorter by 58% among patients who stay comparatively close (≤5 km) to primary care facilities (aOR = 0.42, 95% CI 0.12–0.90; *p* < 0.05) compared to those who stay further away. Pre-hospital delay was also shorter by 85% among patients who experienced chest pain (aOR = 0.15, 95% CI 0.05–0.48; *p* < 0.01) compared to those who presented with symptoms other than chest pain. Pre-hospital delay was reported to be 17.81, 4.45, 98.67 and 2.65-folds longer among patients who did not consider symptoms to be serious (aOR = 17.81, 95% CI 5.92–53.48; *p* < 0.01), were referred from government hospital (aOR = 4.45, 95% CI 2.03–9.74; *p* < 0.01) and private hospitals (aOR = 98.67, 95% CI 11.87–820.34; *p* < 0.01), and without family history of MI (aOR = 2.65, 95% CI 1.24–5.71; *p* < 0.05) compared to patients who were suspected to have MI, were directly admitted to RMCH, and with family history of MI respectively. The adjusted *R*^2^-value (0.528) showed that our selected model could explain the variation of outcome variables by 52.8% (Table 3).

**Table 3:** Characteristics associated with prolonged pre-hospital delay in patients with myocardial infarction in multivariate logistic regression

Characteristics	aOR (95% CI for aOR)	*P*-value
Age category		
≤ 40 years Vs > 60 years^R^	2.43 (0.73–8.12)	0.149
41–60 years Vs > 60 years^R^	0.44 (0.21–0.93)	0.032
Family income		
Lower Vs Higher^R^	5.74 (0.89–37.06)	0.066
Middle Vs Higher^R^	14.22 (2.15–94.17)	*p* < 0.001
Residence		
Rural Vs Urban^R^	1.78 (0.73–4.34)	0.204
Distance of primary care center		
≤ 5 km Vs > 5 km^R^	0.42 (0.12–0.90)	0.026
Predominant clinical symptom		
Chest pain Vs Symptoms other than chest pain^R^	0.15 (0.05–0.48)	*p* < 0.001
Behavior after onset of symptoms		
Misinterpreting the nature of pain Vs Suspected as MI^R^	1.46 (0.47–4.59)	0.515
Did not consider the symptoms to be serious Vs Suspected as MI^R^	17.81 (5.92–53.48)	*p* < 0.001
First medical action after onset of symptoms		
Self-medication Vs Visiting qualified doctor^R^	1.50 (0.42–5.37)	0.534
Visiting non-qualified practitioner Vs Visiting qualified doctor^R^	1.03 (0.43–2.48)	0.943
Mode of admission		
Referred from government hospital Vs Direct admission^R^	4.45 (2.03–9.74)	*p* < 0.001
Referred from private hospital Vs Direct admission^R^	98.67 (11.87–820.34)	*p* < 0.001
Medical history/ risk factors		
Diabetes mellitus (No Vs Yes^R^)	1.02 (0.48–2.16)	0.961
Previous history of chest pain (No Vs Yes^R^)	1.15 (0.53–2.47)	0.727
Family history of MI (No Vs Yes^R^)	2.65 (1.24–5.71)	0.012

*R* Reference case

### In-hospital mortality

A total of 49 patients recruited in our study died during the hospital stay (in-hospital mortality rate 14.5%). Among them, 41 patients (83.7%) had pre-hospital delay of longer than 6 h. The adjusted *R*^2^-value demonstrated that the model could explain the outcome variable by 30.3%.

Multivariable logistic regression model showed that risk of death was lower among adult patients by 75% (aOR = 0.25, 95% CI: 0.12–0.52; *p* < 0.05) compared to aged patients. We also noted that the risk was lower by 72% among patients who were admitted into hospitals earlier (pre-hospital delay ≤6 h) (aOR = 0.28, 95% CI 0.12–0.66; *p* < 0.05) compared to those with pre-hospital delay of more than 6 h (Table 4).

**Table 4:** Determinants of in-hospital mortality of myocardial infarction patients (*n* = 337)

Characteristics	Survival	Death	cOR	aOR
Age category				
≤ 40 years	47 (100.0)	0 (0.0)		
41–60 years	167 (92.8)	13 (7.2)	0.16 (0.08–0.32)*	0.25 (0.12–0.52)*
> 60 years^R^	74 (67.3)	36 (32.7)		
Sex				
Male	213 (83.9)	41 (16.1)	1.81 (0.80–4.02)	
Female^R^	75 (90.4)	8 (9.6)		
Predominant clinical symptom				
Chest pain	227 (86.6)	35 (13.4)	0.67 (0.34–1.33)	
Symptoms other than chest pain^R^	61 (81.3)	14 (18.7)		
Behavior after onset of symptoms				
Misinterpreting the nature of pain	60 (89.6)	7 (10.4)	0.72 (0.29–1.76)	
Did not consider the symptoms to be serious	55 (82.1)	12 (17.9)	1.34 (0.62–2.87)	
Waited to see symptoms would going	32 (82.1)	7 (17.9)	1.34 (0.53–3.40)	
Suspected as MI^R^	141 (86.0)	23 (14.0)		
Diagnosis				
STEMI	209 (83.3)	42 (16.7)	2.27 (0.97–5.26)	
Non-STEMI^R^	79 (91.9)	7 (8.1)		
Pre-hospital delay				
≤ 6 h	125 (94.0)	8 (6.0)	0.25 (0.11–0.56)*	0.28 (0.12–0.66)*
> 6 h^R^	163 (79.9)	41 (20.1)		
Risk factors				
Smoking				
No	114 (87.0)	17 (13.0)	0.81 (0.43–1.53)	
Yes^R^	174 (84.5)	32 (15.5)		
Sedentary lifestyle				
No	191 (84.5)	35 (15.5)	1.27 (0.65–2.47)	
Yes^R^	97 (87.4)	14 (12.6)		
Diabetes				
No	203 (85.3)	35 (14.7)	1.05 (0.53–2.05)	
Yes^R^	85 (85.9)	14 (14.1)		
Hypertension				
No	103 (92.0)	9 (8.0)	0.40 (0.19–0.87)*	0.49 (0.22–1.12)
Yes^R^	185 (82.2)	40 (17.8)		
Previous history of cardiovascular disease				
No	221 (87.0)	33 (13.0)	0.63 (0.32–1.21)	
Yes^R^	67 (80.7)	16 (19.3)		
Family history of myocardial infarction				
No	178 (80.2)	44 (19.8)	5.44 (2.09–14.13)*	1.88 (0.66–5.38)
Yes^R^	110 (95.7)	5 (4.3)		

*R* Reference case

*p-value < 0.05

## Discussion

One of the aims of this study is to identify the determinants of pre-hospital delay for MI patients in the Northern region of Bangladesh. By knowing the factors that may influence the time spent before hospital admission after onset of MI, we may help to formulate strategies to reduce the delay in initiation of effective life-saving treatment for this condition.

The median pre-hospital delay of our study population was 9 (IQR 13) hours, while the median decision time was 2 (IQR 2.8) hours. Referral time from private hospitals was noted to be longer than that of the government hospitals. Overall, only 39.5% of our patients were admitted to RMCH within 6 h of their symptom onset. Patients who had typical clinical presentation, suspected the symptoms as cardiac in origin, visited qualified doctors, and seek treatment directly with RMCH were more likely to be admitted within 6 h after onset of symptom.

A study conducted in a Dhaka, the capital city of Bangladesh, reported that the mean pre-hospital delay for their MI patients was 11.67 h, and about 77% of the them reached the hospital within 6 h [15]. That study was conducted in private tertiary care hospital, where most of patients were from affluent families. Patients from high income families in our study also showed shorter pre-hospital delay compared to others from lower- and middle-income families. Another study conducted in a government tertiary care hospital situated in Chittagong in Southern part of Bangladesh [16] reported early presentation (within 6 h) in 17.2% of their patients. In Bangladesh, the set up and facilities of all government tertiary care hospitals were quite similar all over the country. We could expect that the main factors that contributed towards the discrepancy would be on the social demographic features and health care seeking behavior between these regions.

In developed countries, national level pre-hospital delay of MI patients was generally low. The Global Registry of Acute Coronary Events (GRACE) study reported more than 70% of patients presented to hospital within 6 h, with a median of 3 h [18]. The pre-hospital delays reported by studies conducted in our neighboring developing countries like India were between 3.0 to 5.2 h, and almost 60% of patients were admitted to hospital within 6 h [19, 20]. Similar studies in Pakistan reported between 66 to 73% of patients presented to hospital within 6 h [17, 21].

In our study cohort, pre-hospital delay for both young and elderly patients was more likely to be more than 6 h. The conventional belief that MI is the disease of the old age influenced many younger patients to attribute some cardiac symptoms to less critical conditions like heartburn or peptic ulcer disease, thus hindered early intervention. Older age had also been noted to be risk factor of pre-hospital delay [16, 18, 22], and this may be due to limited resources and problem with transportation. There was no significant difference in pre-hospital delay between both the genders in our study, and this was consistent with most other studies from both developed and developing countries [10, 16, 23–25]. However, some studies identified female gender as one of the predictors of pre-hospital delay [12, 26].

Patients from rural areas and lower income families were more vulnerable to delayed in hospital admission, though it might not be related with their educational qualification. Previous studies have reported that residence from rural areas [23, 27] and those from lower socio-economic backgrounds [28] were at higher risk of delayed hospital admission due to lack of financial resource and availability of transportation. In addition, those staying closer than 5.0 km from primary care facilities were more likely to reach the hospital within 6 h after onset of MI symptoms. Similar finding had been reported where patients attributed long distance from primary care facilities as the main reason for their late presentations [23, 25, 29]. However, our study showed that the mode of transportation (ambulance or general vehicle) was not associated pre-hospital delay. Interesting, a study from South India reported that patients who used private ambulance had shorter delay compared to those who used public ambulance or other types of transportation [27].

We were not able to show any association between status of education and pre-hospital delay. There were conflicting information in the literature, where some studies reported higher educational qualification reduces the pre-hospital delay [12, 23, 28], while others indicated no relationship between the two [27, 30]. We observed an interesting finding where patients in the middle-income group had a much higher odd for delay in hospital admission than those from the lower-income group. We were not able to explain these findings, and further study into this phenomenon is probably indicated.

We noted that clinical symptoms at the onset of MI were a significant predictor for delay in hospital admission. Patients who presented with chest pain were more likely to be admitted for treatment within 6 h of onset of MI, compared to those with other pain over other sites, or atypical and vague symptoms, and this was also reported by other studies [18, 28]. Patients tend to misinterpret the atypical symptoms to be of non-cardiac in origin, and this would hindered them from seeking urgent medical attention. Although Non-ST-elevated Myocardial Infarction (Non-STEMI) has been known to present with atypical symptoms, which can affect the pre-hospital delay of some patients, they did not influence the pre-hospital delay in our patients.

Patients’ behavior and primary action after onset of symptoms were important factors that closely associated with pre-hospital delay. Patients who suspected they had MI were more likely to have early hospital admission while those who considered the symptoms as nothing serious, or decided to wait for spontaneous resolution were more likely to have delayed hospital admission. This finding was similar to other studies where perceived susceptibility to MI was associated with shorter pre-hospital delay, while misinterpretation of symptoms or pain resistance behavior were associated with longer pre-hospital delay [31, 32]. Visiting non-qualified or under-qualified medical practitioners, consulting drug sellers or self-medication significantly increased pre-hospital delay. Though this practice is rare in developed countries, these behaviors markedly increased the pre-hospital delay [33, 34]. Other studies have reported that even visiting general practitioners would increase pre-hospital delay [19, 25].

Although the patients admitted directly to RMCH had a longer median decision to hospital arrival time, most of them were able to reach the hospital within 6 h. This difference is obvious as most of the patients who have visited government primary health centers were from the rural areas whereas those who were admitted directly to RMCH were mainly from nearby urban areas. The finding was consistent with previous studies from different countries [11, 25, 34]. We did noted another study from Bangladesh that reported the opposite finding, where patients who visited primary care centers were more likely to present early [16]. However, their definition of early presentation was 12 h after onset of symptoms. Our study noted that longer referral time for patients from private hospitals compared to that of government hospitals. It was possible that in private setting, there was a higher tendency for doctors to re-establish the diagnosis before they send the patients off to another institution. Although the admission time to other primary care centers (mean of 1.0 h) was shorter than admission time to RMCH (mean of 1.5 h), this might not confer any benefit for the patients since no reperfusion therapy was offered. In fact, the referral time (mean of 4 h) contributed to the overall delay in admission to RMCH (mean of 9.0 h), and this could have increased the rate of poor outcome since optimum result of reperfusion therapy is time-dependent [6].

Influence of long-standing co-morbidities on pre-hospital delay remains uncertain. Our study shows that patients with diabetes mellitus or previous history of coronary arterial diseases were more likely to be admitted earlier compared to their counterparts, but no positive correlation was noted for those with hypertension. Some studies reported that patients with diabetes mellitus and previous history of cardiovascular disease were associated late presentation [18, 35]. On the other hand, our study showed that positive family history of cardiovascular disease was associated with early presentation to the hospital, and this was consistent with other studies [16, 36]. Perhaps in our population, patients and family members with chronic diseases were generally more health conscious and more familiar with available resources, and these would influence them to seek medical attention early.

In-hospital mortality was noted to be higher among the patients who were admitted after 6 h, even after adjusting for other potential confounding factors like age, sex, type of MI and other comorbidities. Similar finding was reported by another study conducted on German population, where unknown or prolonged pre-hospital delay was associated with increased in-hospital death [37]. Other studies has also shown that treatment efficacy of MI decreases with time after onset of symptoms, and this would increase the risk of mortality [2, 6, 7]. However, longer pre-hospital delay has been shown to be associated with other complication for those who have survived the acute episode of MI [37], but these were not assessed in our study.

Our study provides a clear insight on various factors that were associated with pre-hospital delay of MI patients in Northern regions of Bangladesh. Patients’ behavior and health seeking actions would increase the pre-hospital delay. Misinformation and wrong care seeking behavior were most likely due to lack of knowledge and awareness about common symptoms of MI, [8]. Public awareness about symptoms of MI should be raised so that misinterpretations can be reduced. Moreover, patients should be encouraged to visit qualified physicians or hospitals and medical practice of non-qualified personnel should be restricted to reduce wrong diagnoses that may proof to be fatal for MI patients. Referral time, especially from private primary care hospitals should be reduced. Providing emergency diagnostic support in the primary care centers like ECG and cardiac troponin tests can reduce the referral time and thereby reduce the total pre-hospital delay. Proper use of ambulance service and the newly launched emergency call number (999) may reduce the delay, and these observations have been reported in other countries like Sweden [25].

### Strengths and limitations

One of the strengths of this study is that the sample population was from the Northern region of Bangladesh, and this covered a large portion of the national population. Moreover, patients’ symptoms, diagnoses and other clinical data were obtained from the medical records that reduced the risk of recall bias. The main limitation of this study is that it does not represent the overall population of the country. This was a cross sectional single-center study which doesn’t reflect cover the whole population sample of the country. The time of onset of symptoms and primary actions were based on patients’ statement gathered during the interview, and there was a possibility of recall bias. Moreover, convenience sampling method may also introduce sampling bias. This study did not include patients who did not reach the hospital, died shortly after admission, or died before they were fit for interview. We did not study the factors contributing to the pre-hospital delays. This information might have allowed us to identify other possible contributing factors that could have been avoided. Moreover, patients who went to other primary centers might not be from the same geographical location as for those who seek treatment directly in RMCH, and the comparison of pre-hospital delays between the two groups may not be accurate. An analysis to find out the predictors of pre-hospital delay as well as in-hospital mortality based on delay as an interval measure would be more informative. Other than death during hospital stay, we did not study other forms of morbidities related to MI or its treatment, which can be an important drawback of the study. Further study including other adverse events is suggested for better understanding of the effect of pre-hospital delay on those adverse events.

## Conclusions

In this study of patients with MI in the Northern regions of Bangladesh, the total pre-hospital delay was considerably long compared to other reports from medical literature. Patients’ health care seeking behavior, contact with non-qualified practitioner and referral from private hospitals had a significant role in delaying their presentation to the hospital. A more comprehensive health care system that includes public education on critical conditions to high-risk population groups and improvement in general health delivery system can contribute towards more effective treatment patients with MI in this country.

## Supplementary information

**Additional file 1.** Questionnaire.

## Data Availability

Patient information and supporting documents are available on request to the corresponding author. Prior approval from the Ethical Review Committee of Rajshahi Medical College, Rajshahi, Bangladesh will be needed to assess these documents. .

## Abbreviations

MI: Myocardial infarction
RMCH: Rajshahi Medical College Hospital
PCI: Percutaneous coronary intervention
STEMI: ST-elevated myocardial infarction
Non-STEMI: Non ST-elevated myocardial infarction
aOR: Adjusted odd’s ratio
CI: Confidence interval
