# Supplementary material for: Pre-hospital delay in patients with myocardial infarction: an observational study in a tertiary care hospital of northern Bangladesh
Source: BMC Health Serv Res. 2020 Jul 9;20:633. doi: 10.1186/s12913-020-05505-x (PMC7346615; doi:10.1186/s12913-020-05505-x)
Supplement: Supplementary file 1 — Additional file 1. Questionnaire. [file 12913_2020_5505_MOESM1_ESM.doc]

**Questionnaire**

**Demographic Data**

1. Patient identifying code: __________
2. Age of the participant: __________
3. Sex of the participant:

- Male
- Female

1. Marital status of the participant:

- Married
- Single
- widowed

1. Educational status of the participant:

- None/primary
- Secondary/Higher secondary
- University graduate

1. Family income of the participant:

- Lower (< BDT 15 000)
- Middle (BDT 15 000 – BDT 30 000)
- Higher (> BDT 30 000)

1. Residence of the participant:

- Rural
- Urban

1. Distance of primary care center from residence of the participant:

- ≤5 km
- >5 km

**Clinical data**

1. Diagnosis of the participant:

- STEMI
- Non-STEMI

1. Predominant clinical symptoms of the participant:
2. .
3. .
4. .
5. .
6. .
7. Medical history/ risk factors

- Smoking
- Sedentary lifestyle
- Diabetes mellitus
- Hypertension
- Previous history of chest pain
- Previous history of stroke
- Family history of myocardial infarction

**Health seeking behavior data**

1. Behavior after onset of symptoms

- Misinterpreting the nature of pain
- Did not consider the symptoms to be serious
- Waited to see symptoms would going
- Suspected as MI

1. First medical action after onset of symptoms

- Visiting qualified doctor
- Visiting non-qualified practitioner
- Self-medication

1. Mode of admission to RMCH

- Direct admission
- Referred from government hospital
- Referred from private hospital

1. Mode of transport to RMCH

- Ambulance
- General transport

1. Time of onset of symptom: __________
2. Time of making decision for seeking medical help: __________
3. Time of first medical interaction: __________
4. Time of admission to RMCH: __________
5. In-hospital outcome of the participant: __________

- Survival
- Death
